# Rodents and humans are able to detect the odour of L-Lactate

**DOI:** 10.1371/journal.pone.0178478

**Published:** 2017-05-25

**Authors:** Valentina Mosienko, Andy J. Chang, Natalia Alenina, Anja G. Teschemacher, Sergey Kasparov

**Affiliations:** 1 School of Physiology, Pharmacology and Neuroscience, University of Bristol, Bristol, United Kingdom; 2 Department of Biochemistry and Howard Hughes Medical Institute, Stanford University School of Medicine, Stanford, United States of America; 3 Max-Delbrück-Center for Molecular Medicine (MDC) Berlin-Buch, Berlin, Germany; 4 Baltic Federal University, Kalinigrad, Russian Federation; Monell Chemical Senses Center, UNITED STATES

## Abstract

L-Lactate (LL) is an essential cellular metabolite which can be used to generate energy. In addition, accumulating evidence suggests that LL is used for inter-cellular signalling. Some LL-sensitive receptors have been identified but we recently proposed that there may be yet another unknown G-protein coupled receptor (GPCR) sensitive to LL in the brain. Olfactory receptors (ORs) represent the largest family of GPCRs and some of them are expressed outside the olfactory system, including brain, making them interesting candidates for non-olfactory LL signalling. One of the “ectopically” expressed ORs, Olfr78 in mice (Olr59 in rats and OR51E2 in humans), reportedly can be activated by LL. This implies that both rodents and humans should be able to detect the LL odour. Surprisingly, this has never been demonstrated. Here we show that mice can detect the odour of LL in odour detection and habituation-dishabituation tasks, and discriminate it from peppermint and vanilla odours. Behaviour of the Olfr78 null mice and wildtype mice in odour detection task was not different, indicating that rodents are equipped with more than one LL-sensitive OR. Rats were also able to use the smell of LL as a cue in an odour-reward associative learning task. When presented to humans, more than 90% of participants detected a smell of LL in solution. Interestingly, LL was perceived differently than acetate or propionate—LL was preferentially reported as a pleasant sweet scent while acetate and propionate were perceived as repulsive sour/acid smells. Subjective perception of LL smell was different in men and women. Taken together, our data demonstrate that both rodents and humans are able to detect the odour of LL. Moreover, in mice, LL perception is not purely mediated by Olfr78. Discovery of further LL-sensitive OR might shed the light on their contribution to LL signalling in the body.

## Introduction

L-Lactate (LL) is produced by all cells in the body. In the central nervous system (CNS) both neurons and astrocytes produce and use LL but the ability to store glycogen makes astrocytes a particularly important source of LL during high energy demand [1, 2], leading to the hypothesis of astrocyte-to-neuron lactate shuttling. According to this hypothesis, astrocytes produce and release LL which can be taken up and converted to glucose by neurons during periods of neuronal activation [3, 4]. It has recently become evident that LL may also act as a signalling molecule via cognate G-protein coupled receptors (GPCR) [5]. One of these, known as HCA1 (previously GPR81), has now been formally recognised as a receptor for LL [6, 7]. HCA1 is mainly expressed in adipose tissue, but low levels were also reported in various parts of the brain [8–10]. Since multiple studies suggest that activation of HCA1 requires very high LL concentrations [8, 9], it is unlikely that it has any function in the brain under physiological conditions. Our recent results pointed to the existence of another LL-sensitive G_s_-coupled GPCR in rodent brain. Acting on this putative GPCR, LL evokes noradrenaline release and noradrenergic neuron depolarisation in the locus coeruleus [11]. These effects of LL are concentration-dependent, blocked by D-Lactate, and do not require LL entry into the neuron. Identification of this LL-sensitive GPCR is still pending, raising interest to any novel GPCR which can potentially mediate LL effects. In humans, there are about 800 GPCRs, approximately half of which mediate olfaction (http://www.guidetopharmacology.org). Activation of olfactory receptors (ORs) leads to cAMP accumulation, similarly to G_s_-coupled GPCRs. Most of the ORs are exclusively expressed in the sensory neurons of the main olfactory epithelium but ectopic expression of ORs has also been reported. For example, in testis, ORs are thought to play a role in sperm chemotaxis [12, 13] but their roles in other tissues, including the brain [14, 15], are completely unknown. One of these ectopically expressed ORs is the mouse olfactory receptor 78 (Olfr78) which has rat and human orthologues (Olr59 and OR51E2, respectively [16, 17]). In the mouse, Olfr78 was detected in the brainstem area postrema and nucleus tractus solitarius [16] and later found in smooth muscle cells in small blood vessels in a variety of tissues [18, 19]. In addition, it is also expressed in type I glomus cells of the carotid body [20], prostate gland [21], colon, ovaries, testis, pancreas, and placenta [22]. Human OR51E2 and mouse Olfr78 dose-dependently responded to acetate and propionate (Olfr78: EC_50_ = 2.35mM for acetate and 0.92mM for propionate; OR51E2: EC_50_ = 2.93mM for acetate and 2.16mM for propionate [18]), and LL (Olfr78: EC_50_ = 4mM [20]). The wide distribution of this receptor implies its role in vital physiological processes other than olfaction. In the kidney, Olfr78 was shown to regulate glomerular filtration rate, renin secretion, and blood pressure [18, 19] while, in the carotid body, it was proposed to play a role in hypoxia detection [20].

Presence of Olfr78 in the olfactory system and its sensitivity to LL suggests that rodents and humans should be able to detect the smell of LL. Surprisingly, this has never been convincingly documented. Moreover, in the PubChem database, information about whether LL has a smell is controversial with the most recent entry being “odourless” (https://pubchem.ncbi.nlm.nih.gov/compound/612#section=Top ).

In the present study, we assessed whether rodents and humans are, in fact, able to detect an odour of LL, and the importance of Olfr78 in LL detection. We show that mice, rats and humans are able to smell LL. Since Olfr78 knockout mice were nevertheless able to detect LL, there must be more than one LL-sensitive OR in that species. Identification of alternative LL signalling mechanisms may shed light onto LL transmission pathways outside the olfactory system.

## Material and methods

### Animals

All procedures in wildtype mice were conducted in accordance to the guidelines from Directive 2010/63/EU of the European Parliament on the protection of animals used for scientific purposes, and the experiments performed in this study were approved by the Senate of Berlin Ethics Committee (Landesamt für Gesundheit und Soziales Berlin (LAGESO); study number G0300/13). The experiments in rats were performed according to the UK Home Office's Animals (Scientific Procedures) Act, 1986 and approved by the University of Bristol ethics review committee (protocol number 30/2875). Experiments performed using the Olfr78 null/reporter mouse line were approved by the Institutional Animal Care and Use Committee (IACUC) at the Stanford University School of Medicine (protocol number 19968).

### Animal maintenance

3 and 8 male C57Bl/6 mice were used for habituation-dishabituation and odour detection tasks, respectively. 7 homozygous Olfr78 null [23] and 9 wildtype littermate control mice on a mixed 129P2/OlaHsd and C57Bl/6J background were used for an odour detection task. All mice were male, 5–9 months of age, group-housed, and maintained under a normal light/dark cycle, with free access to food and water.

12 male Lister Hooded rats weighing between 200 and 240g (Harlan) were used in an odour-reward associative learning task. All rats were handled and weighed daily, and maintained on a reversed 12-hour light-dark cycle with free access to food and water. 7 days after the arrival animals were put on a restricted diet for 10 days during which they received seven to nine food pellets per day to maintain their body weight at ~85% of their freely feeding weight. Such a diet regime was maintained as well during training and testing days. The task was performed during the dark phase.

### Odour detection test in mice

In order to test the ability of wildtype mice to detect the smell of LL, we subsequently introduced peppermint (40μl of freshly prepared water (distilled) extract from peppermint leaves), LL (40μl of freshly prepared solution in distilled water, 1M, pH = 7.4, L1750 Sigma, ≥98% purity), and urine (collected at the day of testing from female mice with which the tested male mice had never been in contact, 10μl). The odour sequence introduced to Olfr78 null mice and controls was water (40μl), LL (80μl of freshly prepared solution in distilled water, 2M, pH~7.4, 71718 Sigma, ≥99% purity), urine (collected at the day of testing from female mice with which the tested mice had never been in contact, 10μl), vanilla extract (40μl of 1:10 solution diluted in distilled water), distilled water (40μl). 2M LL was chosen for further testing in Olfr78 null mice and their littermates since 2M LL induced similar behaviour responses in control mice on a mixed background in comparison to responses evoked by 1M LL in C57Bl6/N mice. 30 mins before testing, the male mouse was placed into a fresh test cage, and then an odour was introduced on a piece of Wattman paper (2.5cm x 2.5cm) in a corner of the cage for 3 mins with a 1 min interval between different odours. The time mice spent sniffing an introduced odorous paper was measured by an observer blind to the tested substances.

### Odour habituation-dishabituation test in mice

This olfaction test was employed to assess the ability to distinguish between an odour of LL and other smells, and an ability to get used to it. 30 mins before testing, the mouse was placed into a fresh test cage. Each test cage was used only once. During the next 10 mins a dry and clean cotton stick was introduced into the cage in order to decrease unspecific sniffing not connected with the odour. LL (freshly prepared solution in distilled water, 1M, pH = 7.4, L1750 Sigma, ≥98% purity) was subsequently tested with peppermint (freshly prepared solution from peppermint leaves) and vanilla (diluted in distilled water 1:100 from vanilla extract), and distilled water as a negative control. Each smell was introduced three times for 2 mins with a 1 min inter-trial interval on a cotton stick which was dipped in the solution of the tested substance. The time mice spent sniffing different odours was measured by a researcher blind to tested substances.

### Odour-reward associative learning in rats

An odour-reward associative learning task in rats was employed to identify whether rats are able to use the smell of LL to find a reward. This test was performed according to [24] with modifications.

An aluminium operant box (80cm x 80cm x 50cm) was used. The interior of the box was spray-painted matt black to avoid the presence of visual cues and to provide enough contrast for the movement-tracking software, Biobserve (Viewer^2^ version 2.2.0.91, BIOBSERVE GmbH, Bonn, Germany). Sponges were all identical, measuring 8.5cm x 7cm x 4cm, with a 2cm hole cut in the centre, and were only used for one testing day with an individual animal. Kellogg’s Froot Loops used in the study were shown to be a good reinforcement in operant tasks [25]. All the sessions were recorded by a video camera positioned directly above the operant box to allow observation during trials and subsequent off-line analysis.

For the experiments, one sponge was impregnated with either test odour of LL (freshly prepared solution in distilled water, 1M, pH = 7.4, L1750 Sigma, ≥98% purity) or control odour of almond (1:100 diluted almond extract in distilled water) just before each trial. Almond was chosen for positive control, as it is known that rats can smell it and seem to have no preference nor aversion to it [26]. The sponge with concealed reward was placed in a different corner of the operant box for each trial according to a predetermined sequence of numbers from a random number generator (1, 2 or 3). The rat was always placed in the same corner (0) of the box, nose facing the wall at the start of trial, and the remaining two corners were occupied by empty sponges impregnated with distilled water. In total 12 rats were used for conditioning training, and each rat was tested with both odours. Half of the animals were first conditioned with almond odour and, following a 5-day break, with LL odour. The other half were conditioned in the reverse order.

During the first training day, rats were allowed to explore the arena for 5 min before a single Froot Loop was placed on the floor of the box to familiarize the rats with the reinforcement and the experimental arena. Rats were left to consume the food and then returned to their home cages, and the procedure was repeated (i.e. two training episodes for each rat). During the second training day, a similar protocol was followed with the exception that three (odourless) sponges were placed in the corners of the box, and during the second trial the Froot Loop was placed into the hole of a sponge to ensure that the rats were able to retrieve the reward in the course of their normal foraging behaviour.

On the testing day a few Froot Loops were crushed to a powder and sprinkled over the floor of the whole box to prevent the rats from being guided by the smell of the hidden Froot Loop during the following trials. Each session consisted of 6 trials. For the first trial only (introductory trial 0), the food reward was placed on the top of the sponge so that the animals could visually locate it and this trial was not included in the analysis. This was necessary because in the preliminary experiments we found that when crushed Froot Loops were spread around the arena, rats essentially never found a concealed reward within a 5 minute period. In the next trials the latency for a correct response (time taken for a rat to retrieve reward) and errors were recorded. Errors were counted as a nose-poke in a water-impregnated sponge, or sniffing at the correct sponge with the odour without a nose-poke afterwards. The same sponges were used for repeat trials with each rat but the position of the target sponge was changed randomly after every trial. After finding the food reward, rats were allowed to eat it before being removed from the box and returned to their home cage. A cut-off time of 5 mins and 2–5 mins of inter-trial interval were maintained.

### Odour detection test in humans

The protocol for this study was approved by the University of Bristol Ethics Committee (approval 47121). A verbal consent to be part of the study was obtained from every participant.

In order to test whether humans can detect an odour of LL, we recruited 94 young female and male volunteers (Table 1), and asked them to indicate the presence of an odour in four jars containing various chemicals or water. The test was performed during three independent days with groups of mixed gender. The order of the odour presentation was random, and the participants were not informed about what kind of odours to expect. 100ml of 1M (pH = 7.4) freshly prepared solutions in distilled water of LL (L7022 Sigma, ~98% purity), pyruvate (P2256 Sigma, ≥99% purity), propionate (P1880 Sigma, ≥99% purity) or acetate (S2889 Sigma, ≥99% purity) were put in wide odourless glass jars, which were kept closed between tests. Distilled water served as control. The participants were given a questionnaire to determine (1) whether they could detect any smell in the jar (yes/no), (2) if they perceived this smell as pleasant, unpleasant, or neutral, and (3) whether they perceived the smell as sweet, sour/acid, bitter, neutral, or “hard to define”. Participants were instructed on how to perform the test and then executed it themselves. Only one participant was present in the test room at a time.

**Table 1:** Key outcomes of the human odour detection test.

			fraction of participants characterised the smell as*		
test substance	age of participants, years	participants detecting the smell	pleasant	unpleasant	neutral
**L-Lactate**	20.1 ± 0.4	92.6% (87/94)	36.8% (32/87)	19.5% (17/87)	43.7% (38/87)
**acetate**	20.5 ± 0.6	95.3% (61/64)	6.6% (4/61)	49.2% (30/61)	44.3% (27/61)
**propionate**	19.9 ± 0.4	98.4% (60/61)	1.7% (1/60)	71.7% (43/60)	26.7% (16/60)
**pyruvate**	20.0 ± 0.4	96.8% (61/63)	52.5% (32/61)	24.6% (15/61)	23.0% (14/61)
**water**	20.1 ± 0.4	9.6% (9/94)	11.1% (1/9)	44.4% (4/9)	44.4% (4/9)

*Percentage of participants who rated the presented odours as pleasant, unpleasant or neutral was calculated from the total number of test subjects who detected an odour in the jar.

### Statistical analysis

Results are expressed as mean ± SEM. Statistical analysis was performed by one- or two-way ANOVA, or repeated measurements ANOVA with Bonferroni's correction as a post-hoc test for multiple comparisons, t-test, or Fisher’s exact test calculated from a contingency table (PRISM, GraphPad, San Diego, CA). To analyse reward-associated learning in rats all the error data were transformed into correct/incorrect binomial data and evaluated by contingency analysis to compare distributions (either chi-square test for trend or chi-square test for goodness of fit). P < 0.05 was considered significant.

## Results

### Mice are able to detect and discriminate the odour of L-Lactate

**Fig 1:**
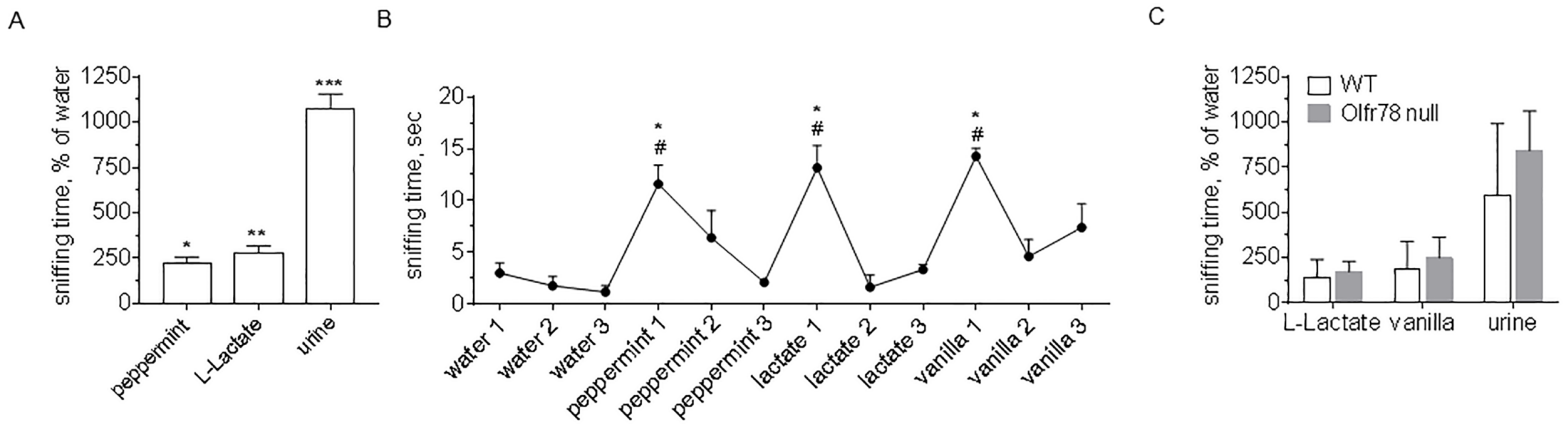
Mice are able to detect and discriminate the odour of L-Lactate. **A. Odour detection task**. Mice spent significantly more time sniffing L-Lactate (1M) than water, and the same amount of time as peppermint extract. Data are shown as mean±SEM, n = 8. *p<0.05, **p<0.01, ***p<0.001 vs water, paired t-test. **B. Odour habituation-dishabituation test**. On the first presentation, mice spent significantly more time sniffing L-Lactate in comparison to water and to the third introduction of the previous odour (peppermint). Over several introductions mice habituated to the smell of L-Lactate and sniffed it for shorter times during the third introduction compared to the first Data are shown as mean±SEM, n = 3. ^#^p<0.05 vs third introduction of previous odour, *p<0.05 vs first water introduction, paired t-test. **C. Odour detection task in Olfr78 null mice**. Olfr78 null mice spent the same amount of time as control littermates sniffing L-Lactate (2M), or control smells–vanilla and urine of the opposite gender. Data are shown as mean±SEM, n_Olfr78 null_ = 7, n_WT_ = 9.

First, we tested in an odour detection task whether mice are able to smell LL. Mice spent significantly more time sniffing LL-impregnated paper compared to water (LL 13.59±1.84 sec vs water 4.84±0.64 sec, p<0.01), which was close to the amount of time they spent sniffing peppermint (LL 13.59±1.84 sec vs peppermint 10.68±1.79 sec, p>0.05, Fig 1A). Mice spent 11 times longer sniffing a paper containing urine in comparison to water (urine 52.12±3.85 sec vs water 4.84±0.64 sec, p<0.001, Fig 1A), and 4 times longer in comparison to LL (urine 52.12±3.85 sec vs LL 13.59±1.84 sec, p<0.001, Fig 1A). This is not surprising because for rodents urine of the opposite gender is one of the most potent odorant signals.

In another behaviour paradigm, we introduced each olfactory stimulus three times (water, peppermint, vanilla, and LL; Fig 1B). Mice had little interest in water but when any of the odours was introduced for the first time, they spent a significant length of time sniffing them (sniffing times: 11.6±1.82 sec for peppermint 1, 13.17±2.15 sec for LL 1, and 14.27±0.78 sec for vanilla 1 vs 3±1.02 sec for water 1, p<0.05). When any of the odorants was introduced repeatedly, sniffing times reduced from the first to the third presentation. For example, for LL differences were as follows: second vs first presentation: 1.63±1.17 sec vs 13.17±2.15 sec, p<0.01; third vs first presentation: 3.33±0.48 sec vs 13.17±2.15 sec, p<0.05. Mice could differentiate the odour of LL from the previously introduced peppermint (2.1±0.15 sec vs 13.17±2.15 sec, peppermint 3 vs LL 1, p<0.05). The smell of LL was also differentiated from the subsequently introduced vanilla as the sniffing time increased again (last presentation of LL 3.33±0.48 sec vs first vanilla presentation 14.27±0.78 sec, p<0.01).

Olfr78 null mice were not different to wild type controls in their ability to detect the odour of LL, and spent similar amount of time sniffing odours of LL, vanilla or urine (Fig 1C). Particularly, Olfr78 null mice spent significantly more time sniffing LL-, vanilla- or urine-impregnated paper compared to water (water 3.87±0.68 sec vs LL 6.67±0.83 sec, p<0.05; vanilla 9.62±1.67 sec, p<0.01; urine 32.56±3.2 sec, p<0.0001).

### Rats can use the odour of L-Lactate as a cue

**Fig 2:**
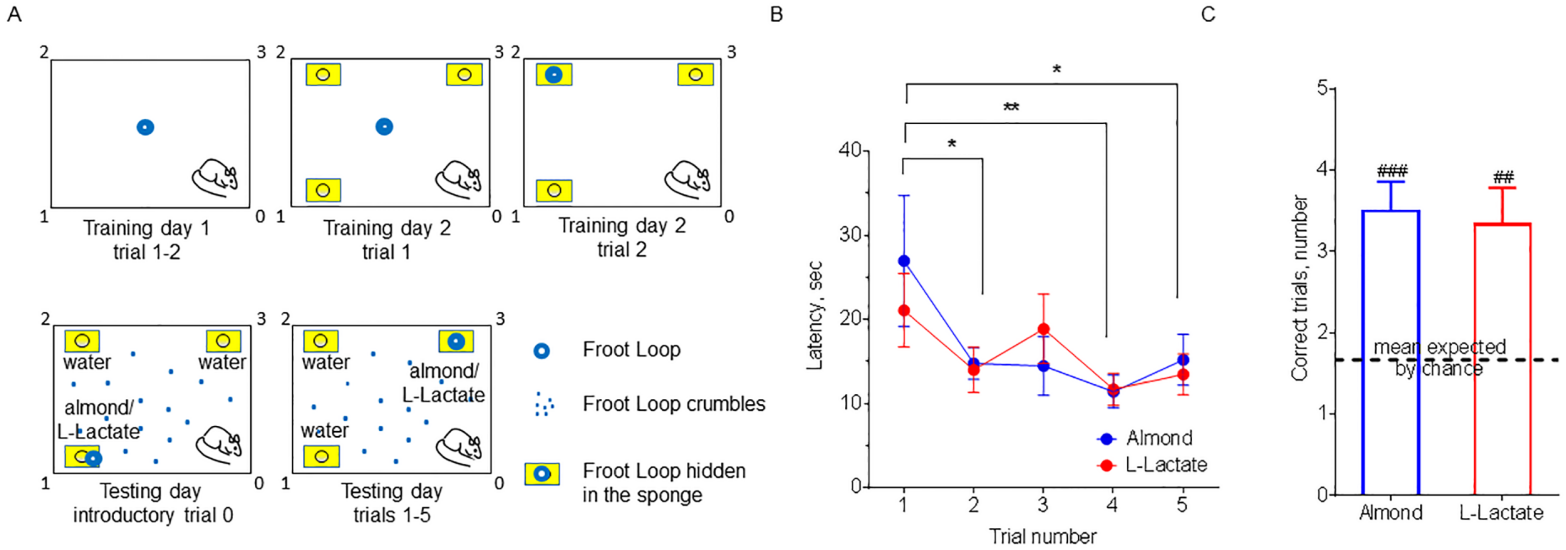
Rats are able to discriminate the odour of L-Lactate. **A. Schematic illustration of an odour-reward associative learning task in rats**. During the training days, rats were habituated to the arena and reward retrieval procedure. During the introductory trial (0) on the testing day (not included in further analysis), a sponge soaked with the test odorant carried a visible food reward. During consequent five trials on the testing day, a randomly placed sponge soaked with the test smell dispensed a hidden food reward. **Outcomes of odour associative learning task in rats: B**. Time taken by rats to find a food reward by the smell (latency) was not different between L-Lactate and a positive control, almond. Over the trials the latency to find a food reward decreased for both odorants. Data are shown as mean±SEM, n = 12. *p<0.05, **p<0.01, two-way ANOVA with Bonferroni’s correction. **C**. Distribution and mean of correct trials for rats did not differ between L-Lactate and almond. Number of observed correct trials was significantly higher than expected by chance. Data are shown as mean±SEM, n = 12. ^###^p<0.001, ^##^p<0.01 vs mean expected by chance, t-test.

To test the ability of rats to detect the odour of LL we employed an odour-reward associative learning task (Fig 2A). During the first test involving a hidden Froot Loop (trial 1), rats took a similar amount of time to find the reward using the smell of LL or almond (21.08±4.36 sec vs 26.98±7.78 sec, LL vs almond, p>0.05, Fig 2B). The following trials (2–5) revealed the effect of trial number on the latency to find the reward by smell (F (4,44) = 4.610, P<0.01, Fig 2B), while the choice of the odorant made no significant difference (F (1,11) = 0.06604, P > 0.05, Fig 2B). During the last trial (5), rats found the reward much quicker than in the beginning (trial 1 vs trial 5, p<0.5, Fig 2B), irrespective of the odorant cue. Next, we assessed how many rats succeeded to find a reward without any incorrect nose pokes. LL was equal to almond in this regard (p>0.05, Fig 2C), and the probability of a correct choice was higher than what may be expected by chance (p<0.001 for almond, p<0.01 for LL, Fig 2C). The probability of randomly chosen correct responses was calculated from the assumption that if the rats do not distinguish an odour of either LL or almond, they would have 1/3 of chance per trial for a correct nose spoke between three sponges. When calculated over 5 trials, the number of randomly chosen correct choices would be 1.67 (Fig 2C). The distribution of chance was modelled using binomial theorem to calculate expected proportion of rats for each number of correct responses. Additionally, a chi-square for goodness of fit tested for a discrepancy between the observed distributions for almond and LL and the expected distribution by chance and returned very low p-values for both (p<0.0001 for both odour groups). Altogether, these data indicate that the odour conditioning enabled rats to correctly identify location of the reward using both LL or almond odours as olfactory cues.

### Humans are able to detect the odour of L-Lactate

Young female and male volunteers were asked to smell jars containing solutions of LL, acetate or propionate which were reported to also activate Olfr78 *in vitro* [18, 20]. Pyruvate was included as a molecule carrying the same caloric value as LL and chemically similar to LL, but which was not previously described as an olfactory stimulant, nor a ligand for Olfr78. 92.6% of the participants indicated the presence of a smell in a jar containing a solution of LL, 36.8% and 19.5% out of which characterised this smell as pleasant and unpleasant, respectively (Table 1). Interestingly, 96.8% of the volunteers detected an odorant in a jar containing pyruvate, again mainly characterising its smell as pleasant (52.5% vs 24.6%, Table 1). To note, the proportion of participants indicating pyruvate as a pleasant odour was higher compared to LL (pyruvate 52.5% vs. LL 36.8%, p<0.0001). Acetate and propionate were characterised as odorous substances by 95.3% and 98.4%, respectively, with a large majority characterising them as unpleasant (Table 1).

**Fig 3:**
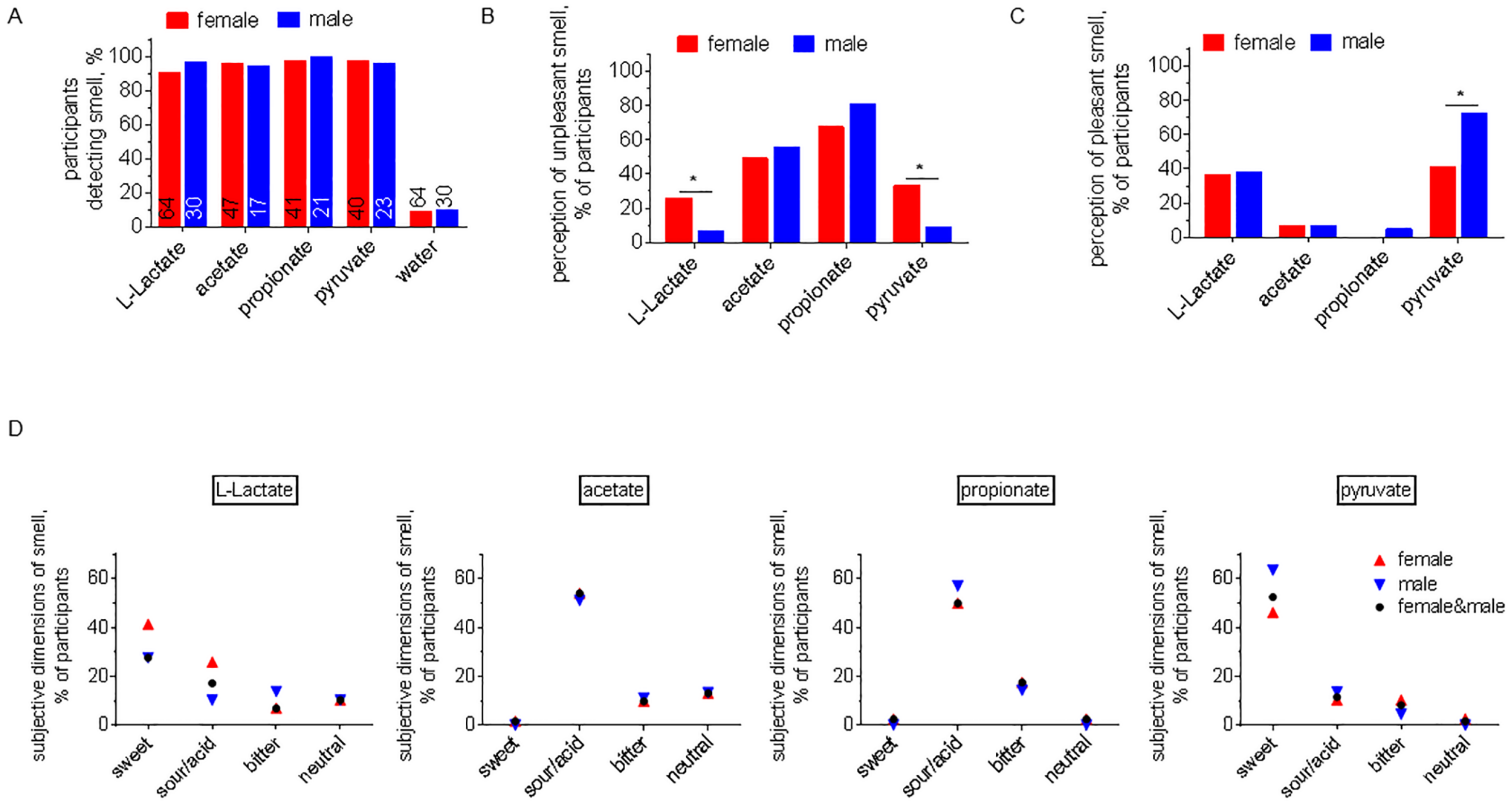
Gender-dependent differences in L-Lactate odour perception by humans. **A. Gender-dependent differences in odour discrimination**. Similar percentages of female and male participants were able to discriminate an odour in solutions of L-Lactate, pyruvate, acetate and propionate. Numbers indicate amount of participants tested with a smell. **B. Gender-dependent differences in ‘unpleasant’ odour perception**. L-Lactate and pyruvate were perceived as repulsive by more women than men (L-Lactate: 25.9% vs 6.9%; pyruvate: 33.3% and 9.1%). Similar percentages of female and male participants rated the odours of acetate and propionate as unpleasant. **C. Gender-dependent differences in ‘pleasant’ odour perception**. Almost twice more men than women rated the smell of pyruvate as appealing (72.7% vs 41%). Similar percentages of men and women rated the odour of L-Lactate, acetate and propionate as pleasant. **D. Gender-dependent characterisation of test odours**. L-Lactate, pyruvate, acetate and propionate were rated similar by men and women in respect to the dimension of their smell/taste. Most participants, independent of gender, indicated them as sweet and sour/acid, respectively. Lactate and water: n(F) = 64, n(M) = 30; acetate: n(F) = 47, n(M) = 17; propionate: n(F) = 41, n(M) = 21; pyruvate: n(F) = 40, n(M) = 23. *p<0.05, Fisher’s exact test.

There was no significant difference between men and women in the frequency of detection of any of the odours (Fig 3A). However, there was a difference in how LL odour was perceived by men and women, since many more women characterised it as unpleasant (25.9% vs 6.9%, p<0.05) (Fig 3B). Curiously, men also did not find the smell of pyruvate as unpleasant as women did (33.3% vs 9.1%, p<0.05). The frequency of reporting LL as a pleasant smell was not gender dependent but for pyruvate the proportion of men liking it was significantly greater (72.7% vs 41%, p<0.05, Fig 3C). Irrespective of gender, odours of acetate and propionate were perceived as an unpleasant by most of the participants (Fig 3B).

Perception of smell is highly subjective. When participants were asked to characterise their perception of the odorants, LL and pyruvate were often described as a sweet substance, while acetate and propionate were mostly characterised as sour/acid (Fig 3D). However, more participants indicated pyruvate odour as sweet in comparison to LL (LL 27.6% vs 52.5% pyruvate p<0.01). Only a few participants (9 out of 94) indicated a presence of odour in a jar containing water which approximates the rate of false detection in this test (p<0.0001 for water vs any other tested odour, Fisher’s exact test, Table 1).

## Discussion

LL is a remarkable molecule which stands out from the myriad of chemicals which surround us in everyday life, because it is pivotal to metabolism of all cells and is present in foods and body secretions [27–30]. After the discovery of LL-sensitive GPCRs in the brain [6, 7, 11], LL was recognised as a potential signalling molecule, raising the interest in any possible receptors for this molecule. In humans, ORs constitute half of the known GPCRs (http://www.guidetopharmacology.org), and represent a big group of potentially LL-sensitive GPCRs some of which are found outside of the olfactory epithelium and probably important irrespective of their role in olfaction. Here, we tested whether LL can serve as an odorant in rodents and humans, and assessed potential contribution of Olfr78 in LL odour perception. To this end, we performed several odour detection or odour-reward associative learning tasks in rodents and humans. In order to delineate the involvement of Olfr78 in sensing LL, we used an Olfr78 null mouse model in an odour detection task.

Here for the first time we show that mice are able to detect the smell of LL and discriminate it from peppermint and vanilla odours. Rats were able to perform an odour-reward associative learning task using the smell of LL as a cue, which worked approximately as well as a standard odorant, almond.

When tested in an odour detection task, Olfr78 null mice had the same behavioural responses as control mice to the smell of LL. This is not entirely surprising because there are only very few cases when an ablation of (or a mutation in) one of the ORs would lead to a change in odour-evoked behaviour. One of such ORs is odr-10 in *C*.*elegans*, ablation of which leads to the loss of chemotaxis specifically to diacetyl, but not to other substances known to cause chemotaxis in this species [31]. Two further examples of such receptors in mice are trace-amine associated receptor (TAAR) 4 and 5, ablation of which resulted in lack of aversion to predator urine [32] or lack of attraction to murine urine [33], respectively.

There is a possibility that the sensitivity to LL was not completely lost but only reduced in Olfr78 null mice. Testing responses to lower LL concentrations in odour discrimination task might help to further clarify this issue.

On the other hand, odour detection and coding is typically combinatorial. The same odour is usually detected by more than one receptor while the same receptor may be responsible for the detection of different odours [34, 35]. Based on the sequence similarity, Olfr558 (human orthologue OR51E1) is assigned to the same subfamily as Olfr78 and it is known that ORs of the same family frequently detect odorants with similar structures [35, 36]. Indeed, it was shown that Olfr558 is activated by propionate [37, 38]. It is therefore possible that Olfr558 might be able to interact with LL and could explain why Olfr78 deletion in mice did not prevent detection of LL. Interestingly, Olfr558 is also expressed outside of the olfactory system [20].

Two other ORs, OR1G1 and OR52D1, were recently shown to be sensitive to propionate [37, 39], but they have not yet been tested for the ability to sense LL. BLAST search using the cDNA sequence of Olr59 (rat analogue of Olfr78, GenBank AY317512.1) as a tag revealed multiple olfactory receptor genes with more than 60% similarity to Olfr78. One such a gene is Olfr406, which is 96% identical to Olfr59 at the DNA level. There is no information on the ligands of this receptor but it might be yet another OR responsible for LL detection in Olfr78 null mice.

When tested in humans, less than 10% of participants were unable to detect the odour of LL. Interestingly, besides LL, the chemically related compounds propionate and acetate were reported to activate the human orthologue of Olfr78. However, subjectively these substances were perceived differently by the participants. Importantly, all chemicals were neutralised and the effects of protons which could be responsible for some aspects of smell perception was therefore minimal. LL was usually qualified as a rather pleasant odour with a sweet scent, and pyruvate which has not been tested as a potential ligand of the ORs described above, had a similar odour profile as LL. It is likely that humans can distinguish between LL from pyruvate since more participants characterised pyruvate odour as pleasant and sweet in comparison to LL odour. However, such difference in perception might also reflect a difference in perceived odour intensity which contributes to less pleasant experience of the odour. Additional tests would be required to prove this point unequivocally.

In contrast, propionate and acetate were predictably characterised as unpleasant (sour/acidic) smells. Various odorants may have different affinities to specific ORs and a slight change in chemical structure or even concentration of an odorant might change its perception. For example the substitution of the hydroxyl group to carboxyl in octanol changes its perception from rose-like to rancid and sweaty [40]. Enantiomers of carvone are perceived either as caraway or spearmint [41]. Even more surprisingly, some chemicals with completely different structures can be perceived as the same odour [42]. Thus, it is likely that recognition of any of the tested chemicals relies on interaction with several receptors in addition to Olfr78 (or its orthologues).

In this study, we did not detect any difference in ability to detect the tested odorants between men and women as similar percentages of both genders indicated the presence of the odour in the jars containing acetate, propionate or LL. However, subjective dimensions of the presented smells were different. Significantly more men than women found a smell of pyruvate pleasant, while more women indicated this smell as repulsive. Interestingly, there was a similar percentage of participants of both genders who indicated the smell of LL as pleasant, however a bigger proportion of women than men indicated it as unpleasant. It was noted a long time ago that women are superior in olfactory perception compared to men [43, 44]. Various experiments established that intensity (a perceived strength of odour sensation) of different odours might be perceived differently by men and women. However, the hedonic valency (pleasant/unpleasant) was rarely tested between different sexes. Whether the gender-dependent difference in odour perception observed in our experiments is influenced by or is due to differences in OR expression, and what the biological impact of such differences may be, is unknown.

### When could LL olfaction be behaviourally relevant?

All chemicals tested here are naturally occurring in humans and animals and they appear in sweat, breath and various secretions [27–30]. LL in combination with ammonium bicarbonate is used in mosquito attractants (Lurex brand). Such a combination is claimed by the manufacturer to mask naturally occurring human skin odours which lure mosquitos closer to the trap where they are more likely to be captured. Furthermore, yellow mosquitos which suck blood mostly from humans choose their host based on the lactic acid smell [45]. LL is not a typical odorant molecule which are usually volatile and lipophilic. In contrast, LL is highly water soluble and not very volatile. It also lacks classical chemical signatures of odorants, such as aromatic rings. It is still unclear whether rodents use the odour of LL by itself or in combination with other substances to perceive and identify certain changes in the environment, their mates or threats. The chemicals studied here had to be used at fairly high concentrations, suggesting that their detection as odorants is only possible during a close contact with the object. Nevertheless, the ability to detect LL smell is evolutionary conserved even though humans have lost a large part of the ORs present in animals. This probably points at the evolutionary advantage of this cue for human behaviour which, so far, has neither been studied nor appreciated.

Responses to olfactory stimuli can be mediated not only by the olfactory system but also through the somatosensory system [46]. The trigeminal system can respond to somatosensory stimulation elicited by an odorant due to the presence of free nerve endings of maxillary branches of the trigeminal nerve in the nasal cavity [47]. Usually sensations perceived by this system are described as burning, stinging, itching, tickling, cooling or warming. Such a response is protective since long exposure to odorants which cause strong trigeminal system activation may result in serious airway damage (pulmonary haemorrhage, dyspnoea, dermal irritation). In anosmic humans lacking olfactory nerve function, propionate and acetate were shown to cause trigeminal responses [48, 49]. The contribution of the somatosensory system to detection of LL and pyruvate remains to be elucidated. However, the biological relevance of such a response is unclear since neither of these substances is toxic.

Changes in odour perception were reported in some psychiatric conditions such as schizophrenia [50, 51] Moreover, a basic smell test is a sensitive method to diagnose the severity of Parkinson’s [52–54] or Alzheimer’s Disease [55, 56]. Whether dysfunction in odorant perception might be used as a screening platform in clinics or whether it can point to the dysfunction of OR signalling in non-olfactory CNS areas still has to be elucidated. However, the importance of discovering LL receptors which are ectopically expressed, should not be underestimated. LL receptor-mediated signalling via different pathways [5] may be important outside the olfactory system. For example, LL signalling via Olfr78 is involved in the detection of hypoxia by the carotid body [20]. Recently, we described LL-evoked noradrenaline release and cell depolarisation in the locus coeruleus, the largest hub of central noradrenaline-producing neurons, suggesting existence of yet another LL receptor [11]. Discovering other LL sensing ORs might help to further our understanding of the signalling role of LL in the brain and offer targets for development of novel drugs.

To summarise, we demonstrate that rodents (mice and rats) detect the smell of LL, as may be expected, given the existence of at least one known LL-sensitive OR. However, Olfr78 which was previously reported to be LL-sensitive appears to be dispensable for LL detection in mice. Humans also clearly detect the LL smell and women much more often than men characterise it as unpleasant smell. Given the documented presence of ORs outside the olfactory system, we speculate that ectopically expressed ORs represent plausible pharmacological targets.
